# Blood Politics, Ethnic Identity, and Racial Misclassification among American Indians and Alaska Natives

**DOI:** 10.1155/2014/321604

**Published:** 2014-02-10

**Authors:** Emily A. Haozous, Carolyn J. Strickland, Janelle F. Palacios, Teshia G. Arambula Solomon

**Affiliations:** ^1^University of New Mexico College of Nursing, MSC 09 5350, 1 University of New Mexico, Albuquerque, NM 87131, USA; ^2^Psychosocial & Community Health, University of Washington School of Nursing, P.O. Box 357263, Seattle, WA 98195-7263, USA; ^3^San Francisco School of Nursing, 2 Koret Way, Room N411Y, P.O. Box 0606, San Francisco, CA 94143, USA; ^4^Native American Research and Training Center, Department of Family and Community Medicine, University of Arizona, 1642 E. Helen, Tucson, AZ 85719, USA

## Abstract

Misclassification of race in medical and mortality records has long been documented as an issue in American Indian/Alaska Native data. Yet, little has been shared in a cohesive narrative which outlines why misclassification of American Indian/Alaska Native identity occurs. The purpose of this paper is to provide a summary of the current state of the science in racial misclassification among American Indians and Alaska Natives. We also provide a historical context on the importance of this problem and describe the ongoing political processes that both affect racial misclassification and contribute to the context of American Indian and Alaska Native identity.

## 1. Introduction

Serious health inequities exist for American Indians and Alaska Natives (AI/AN) in the United States (US). Previous authors have documented the disparities in health data for this population that are related to disease incidence and mortality rate as complicated by misclassification of race and ethnic identity [1–5]. The biomedical-epidemiologic model that guides our federally funded research paradigm in the US requires the researcher to justify the need for a study based on evidence of population health status through disease incidence and mortality rates, as well as provide the justification of how the study will contribute to the advancement of science.

In a research climate in which epidemiologic evidence is critically important, misclassification of race is of special concern for AI/ANs, who make up only 5.2 million or 1.7% of the US population [6]. With such a small population size, AI/ANs often are dropped from analysis for lack of statistical significance, omitted from national reports, and subsequently overlooked as recipients of needed resources. Yet this is a population with documented health disparities, including lower life expectancy and disproportionate poverty rates, nearly three times the rate of type 2 diabetes-related deaths, 1.7 times the rate of suicides and 6.5 times the rate of alcohol-related deaths compared to non-Hispanic Whites, justifying inclusion in reports in spite of the smaller overall proportion of the US population [7]. Recognizing that the actual burden of disease in AI/AN communities is often underreported due to racial misclassification only adds to the importance of this problem.

State surveillance data in the fields of cancer [1], sexually transmitted infections [8], cardiovascular disease [9] and death [10, 11] indicate not only AI/ANs are often misclassified, but actual health disparities figures are also larger than reported. When examined, two overall issues which contribute to the majority of the misclassification are (a) human error and (b) system failures. Researchers have highlighted the extent of the problem and explored tracking and linkage methods to address misclassification [1, 3, 5, 11]. Others have examined the social processes and conditions under which misclassification occurs [2, 4, 12]. While progress has been made in addressing this area of health disparities for AI/ANs, much more is needed. The purpose of this review is to revisit the problem of racial misclassification among AI/ANs, provide a historical context for understanding misclassification, and offer solutions to improve reporting on this population. It is expected that the information and discussion presented will benefit those researchers, policy makers, and funding agencies working with AI/AN communities, with implications for those working with other diverse or vulnerable populations. This examination will take place in the context of critical race theory, used here as a tool to analyze the power structure underlying the forces that have created systematic racial misclassification.

## 2. American Indian and Alaska Native Identity

American Indian and Alaska Native tribal affiliation guidelines are varied and often based on complex tribal histories and sociopolitical processes which have led to multiple terms and levels of AI/AN identity. In addition, Tribes, Pueblos, and Nations can be federally recognized, state recognized, and unrecognized by either state or federal government, and people can self-identify as being AI/AN. To date, there are 566 federally recognized tribes [13] and 66 state recognized tribes [14].

### 2.1. American Indian History

To best understand AI/AN ethnic identity, knowledge about AI/AN history is critical as it provides the necessary context to present day conditions such as health behaviors and disease, socioeconomic status, and ongoing marginalization [15, 16]. Since contact with Europeans in 1492, the indigenous peoples of the Americas have systematically faced barriers to their ability to thrive. In particular, the AI/AN people of the US have experienced loss of ancestral land, forced removal to reservations or other lands not originally used, genocide, ethnocide, warfare, disease, starvation, imprisonment for practicing traditional beliefs, sterilization campaigns targeting women, and assimilation methods such as boarding schools and relocation to urban areas [17–19].

As colonial expansion proceeded westward, tribes were forcibly pressured to move from ancestral lands. Many tribes were offered treaties in exchange for ceding their land in government-to-government agreements; most of these agreements were signed between 1817 and 1871. Tribes, which signed treaties with the federal government, ceded traditional lands and resources in exchange for smaller, often distant reservation land and access to health care and education. Only those tribes that signed treaties were federally recognized and given access to resources, leaving those tribes that did not sign to be disenfranchised politically and socially [20, 21]. Members of federally recognized tribes have had legal rights to health care established by treaties, case law, the Snyder Act of 1921 (P.L. 83-568), the Indian Health Care Improvement Act, H.R. 1167, and the Patient Protection and Affordable Care Act (P.L. 111-148) [22].

During the formation of reservations, the federal government developed a solution to acculturating American Indians through the Western values of land ownership and farming. The Dawes Act of 1887 (also known as the General Allotment Act) formalized the allotment of reservation land, enabling the US government to parcel land out to male heads of households. During the allotment period between 1887 and 1934, the term “blood quantum” was officially integrated into the legal status of native identity for the purpose of dividing reservation land into individual allotments [21]. Reservation-bound male heads of households received allotments, though in many cases a 1/4 American Indian blood quantum was used to determine who was eligible for an allotment of land. Due to the blood quantum qualification, many people were ineligible, thereby effectively reducing the Indian holding of land. “Surplus” reservation land was then open to homesteaders and corporations for purchase, creating patchwork quilt-like reservations with both American Indian and non-American Indian land holdings [21]. Over 90 million of the 138 million acres originally designated as Indian territory were lost, and thousands of AIs were displaced [23].

After the allotment period the federal government mandated that every federally recognized tribe determines criteria for tribal enrollment. The federal government suggested using blood quantum and often provided a step-by-step guide for how tribes could determine blood quantum and tribal enrollment [21]. Although each federally recognized tribe was able to determine their own criteria for tribal enrollment (and therefore access to resources), many tribes adhered to a predetermined blood quantum policy. Today, some tribes like the Cherokee Nation have no blood quantum requirement but instead rely upon direct lineage from one ancestor who was listed on the original tribal enrollment of the Dawes Act [24], while the Northern Ute Tribe in Utah requires the highest blood quantum of 5/8 [25].

Present day resources tied to AI/AN enrollment can be credited to the 1934 Indian Reorganization Act (also known as the Howard-Wheeler Act), which encouraged tribes to form their own governments as they moved to self-determination [21]. In addition to abolishing the use of allotments, tribes were allowed to reorganize themselves into corporations, establish a system of credit, receive preferential employment with the Bureau of Indian Affairs (BIA), access support in stabilizing their governments, and receive educational and technical training supported by the federal government. However, the government established a blood quantum of at least 1/4 Indian blood, (composed of any combination of federally recognized tribes) in order to access these benefits. In current times, with adequate proof of ancestry the BIA will issue a “certificate of degree of Indian blood” that does not confer enrollment into a tribe but does give the bearer access to BIA resources [21]. Benefits can be accessed by enrolled members of federally recognized tribes, even if an individual does not meet the 1/4 degree of Indian blood requirement.

### 2.2. Further Complications

To complicate the issue of enrollment further, some tribes, like the Confederated Salish and Kootenai Tribes of Montana, have narrowed their criteria for enrollment (from being born within the reservation border to an enrolled parent to having at least 1/4 degree of Salish/Kootenai blood), effectively splitting families in half of those siblings born before the enacted criteria who were enrolled and those born after the new rule who are not enrolled, creating mixed-status households [26].

Some people may carry 100% Indian blood but not have enough blood of any one federally recognized tribe to be enrolled in a tribe, but they would likely qualify for the certificate of degree of Indian blood. Other tribes have rules regarding patrilineage or matrilineage so that a person may be of sufficient blood quantum with blood quantum tallied from both parents, but not be an enrolled member of a tribe due to rules of patri- or matrilineage. These unenrolled children born to enrolled members of federally recognized tribes are called “descendants” and are often eligible to receive certain benefits (e.g., health care from Indian Health Service) until they are 19 [27]. These children may still be recognized by the larger AI/AN community due to a shared cultural identity and kinship relations. However, they are not considered tribal members and cannot receive Indian Health Service (IHS) as adults nor benefit directly from tribal services which may include academic scholarships, housing, and voting in tribal elections. For example, upon turning 19, a descendant has their IHS health benefits terminated for not meeting tribal enrollment criteria despite inheriting the same health risks and behaviors while sharing the same AI/AN cultural practices.

Additionally, linking to tribal health systems may jeopardize accurate data collection. Under special conditions the following individuals are potentially able to receive IHS or contracted health benefits: (a) non-AI/AN members living in an AI/AN's household, (b) non-AI/AN woman pregnant with an eligible AI/AN's child, (c) any foster child, natural, adopted, stepchild, legal ward, or orphan of an eligible AI/AN, and (d) individuals with close economic and social ties to a particular federally recognized tribe, and so forth [27]. These special circumstances that allow non-AI/ANs access to IHS services further compromise IHS health data. Furthermore, with the decentralization of healthcare to Tribal Health Systems by the PL 93-638 contracts, the lack of uniformity across health systems and medical records systems provides special barriers to linkages for cross-referencing for the purposes of verifying race.

### 2.3. Racialized Identity

Tribes that are neither federally recognized, nor state recognized may petition for state or federal recognition. While not all states have a process for seeking tribal recognition, criteria for recognition vary by state [28]. For example, at one time Virginia had a lengthy and rigorous process for gaining state recognition but now includes legislative appeal which has no policy or set of rules for recognition [29]. Currently, the process of becoming federally recognized is strenuous. Since 1978, the law requires tribes seeking federal recognition to apply to the BIA's Office of Federal Acknowledgment and meet specific criteria regarding community identity, location, and lineage [28]. In a 24-year period between 1978 and 2012, only 352 AI/AN groups have sought Federal recognition. Of this number, only 87 groups have submitted completed petitions, of which 17 have been federally recognized by the Indian Affairs of the Department of the Interior, 19 have been resolved and recognized by Congress or other means (e.g., most commonly they merge with other applicants) and now have federal recognition, 33 groups have been denied recognition, and the remaining 18 groups are in various stages of negotiation and consideration [30, 31].

The racialized AI/AN identity classification derives from an inherited view that phenotypical characteristics that are genetically determined equal an ascribed classification, such as American Indian or Alaska Native. No other US population must persevere through such sociopolitical hoops in order to claim an identity. Federal policies have created the racialized AI/AN identity, and federal policies continue to enact a racialized AI/AN identity through strict requirements to obtain federal recognition and services.

Regardless of legal status, often the most relevant and important designation for an AI/AN individual is their personal sense of tribal affiliation, which manifests itself in self-report. In an age of growing interracial marriages, ethnic identity becomes a complicated math equation with individuals belonging to several tribal groups by lineage but often are raised in one or another ethnically. Urban areas (often defined as population areas greater than 100,000 people) create the perfect example of individuals enacting their tribal affiliation and AI/AN identity. The term “urban Indian” is loosely defined as those who self-identify as AI/AN, reside in an urban area [32], and may or may not include peer judged criteria specific to ancestry, appearance, cultural knowledge, and Indian community participation [33]. There is an unspoken implication that while an individual may self-identify as AI/AN, the urban Indian community may or may not recognize that individual as being “Indian.” Additionally, the term “urban Indian” not only signifies more than a place of residence, but also implies an experience inherited from a time when some federally recognized tribal members were relocated to cities during the late 1940s to early 1960s through the federal relocation program aimed at assimilation, often leaving families stranded without promised resources in cities they did not know, helping create a “Pan-Indian” culture where members of different tribes interacted creating a broader AI/AN identity [32, 33].

One last, albeit important, concern regarding identity is the issue surrounding tribal affiliation and intermarriage. Today it is not uncommon for AI/ANs to have parents from multiple tribes. Currently, most AI/AN tribes require that parents decide on one tribal affiliation for their child, and it is from that tribe that this person will carry official membership from birth forward, although their identity may be from multiple tribes. If a tribe has a blood quantum requirement and intermarriage makes that child ineligible for membership in the tribe, it is possible for a child to have descendance from multiple tribes but no true legal tribal affiliation and remain ineligible for BIA and IHS benefits.

The multiple complex issues resulting from determining AI/AN identity and, therefore, population are at last bound to the significance of numbers. From determining the prevalence of a health problem such as cancer to identifying the total AI/AN population in the US, all relies upon the complicated interactions of how one is classified and how an individual self-reports their racial or ethnic identity. While the public will often attribute heritage or ancestry as a racial identity [34], they also engage in cultural practices that may exhibit multiple ethnic identities.

Recently, researchers have turned their focus to urban AI/AN problems, as this group has high rates of health disparities compared to the general population and sometimes in comparison to their tribally based counterparts [32, 35]. It is common for researchers working with urban AI/AN populations to rely upon self-identification, usually recruited from AI/AN urban health centers and community organizations. Similar to the issues of AI/AN tribal membership, there is no one standard for being an “urban Indian” [35].

## 3. Misclassification

### 3.1. Terminology Defined

In many instances there is confusion between race and ethnic identity [36]. Race has historically been defined based upon physical characteristics (hair color, eye color, height, eye shape, skin color, etc.) and arose during European imperialism and colonization wherein scientific observations often reflected the political relations Europeans had with peoples from different political and cultural traditions [36, 37]. During the early 19th century, race presumed shared biological or genetic traits and was thought to be linked to intelligence, health, and personality. Race as a demographic category is being used widely in contemporary research contexts for demographic purposes, but historically race and racial categorization were used for much more damaging purposes. The “one-drop rule” directed state policy regarding treatment of people of African ancestry and other minorities under the Racial Integrity Act (Virginia SB 219, 1924), effectively promoting a proeugenics agenda in the state of Virginia, although laws prohibiting interracial marriage had existed throughout the continent since the Colonial era [38]. It is now widely accepted that race is a socially constructed term, reflecting the scientific and sociopolitical climate from which it originated to describe biology.

In contrast, ethnicity relates to cultural factors such as language, religion, beliefs, practices, ancestry, and nationality that people within a specific community share. Ethnicity is understood to be a dynamic process combining past and present influences [39]. Ethnicity is also socially constructed and an individual may change their self-report of ethnicity on a day to day, situation to situation basis [40]. In addition, ethnicity is the product of both what an individual *feels* and what others *ascribe* to that individual [39]. AI/ANs share an ethnicity due to broad set of similar beliefs and cultural practices, which have been the target for colonization and marginalization. Within tribes, AI/ANs have specific ethnic identities that are unique to their tribe and culture. For the purposes of this paper, the term “race” refers to the historical term used to classify people based on physical characteristics, while the term “ethnicity” refers to the socially constructed term for an individual or group's cultural identity.

As this paper has outlined earlier, the terms race and ethnicity are frequently used interchangeably. Often health scientists report race and ethnicity as variables, but they are often using race and ethnicity as proxies for a mixture of genetic, biological, environmental, and social factors [40, 41]. While an article by the Journal of the American Medical Association has attempted to improve the reporting of these factors by implementing a policy and procedure for authors; many researchers continue to use race as a proxy for genetics, when in fact their use of race is more closely related to cultural features (ethnicity), socioeconomic status, or education [42].

Ethnicity is slowly replacing race in the scientific literature, and although editorials calling for the addition of ethnicity as a medical subject heading (MeSH) within the National Institutes of Health Library of Medicine (known as PubMed) have been published as early as 2002, ethnicity is still missing as a MeSH term [43]. Additionally, in the field of cultural competence, researchers often miscategorize patients according to their racial identity rather than ethnic identity when trying to understand cultural effects on health care beliefs and practices [44]. In the biomedical world, race and ethnicity are also used interchangeably, but there is a growing movement within genomics in particular to standardize terms so that race reflects geographical, ancestral, or population specific constructs for which environmental effects may be determined [45].

Obfuscating the problem while the scientific community makes efforts in standardizing the terms “race” and “ethnicity,” federal agencies do not follow the same terminology. Since 1997, federal agencies have used a minimum of five race categories: White, Black or African American, American Indian or Alaska Native, Asian, and Native Hawaiian or Other Pacific Islander as required by the Office of Management and Budget to describe subgroups of the population [46]. Historically, the Office of Management and Budget employed the term “ethnicity” to identify ancestral origin (e.g., country of origin), but scientists argue that census responses actually reflect one's self-identity, rather than ancestry [34].

Misclassification of both racial identity and ethnic identity are common and create problems in reporting morbidity and mortality [47]. In particular, state surveillance data often contribute to inaccuracies in characterizing health disparities among minorities, especially among AI/ANs [8]. Even in a private insurer closed electronic system, racial misclassification frequently occurs, especially with minority populations [47].

### 3.2. Unraveling Causal Factors in Misclassification

Reasons related to disparities in health status data and racial misclassification are complex and related to a number of factors for the AI/AN population that include (a) the system of care, (b) methods used in calculating disease rates, (c) limitations in data tracking systems, (d) political processes that redefine tribal enrollment, (e) stereotyping, and (f) systems that do not collect data.  Table 1 provides reasons for misclassification specific to AI/ANs [2, 12, 48].

When linking health care records, it is critical to note that AI/ANs may interface with health care through multiple and varied systems. Indian Health Service clinics and hospitals, urban clinics, and Tribal Health Systems, which serve approximately 2.2 million AI/ANs, are often access points which require the patient to qualify based upon an established and documented heritage [27, 49]. It is important to note that both geographical (e.g., distance) and socioeconomic (e.g., lack of transportation) barriers prevent some AI/ANs from accessing IHS services, despite qualifying for this service. In addition, AI/ANs use Medicaid, Medicare, long and short term care facilities, urgent care facilities, and the Veterans Administration health care system [50]. Job-based or other private health insurances are used by about half (49%) of AI/AN people, while 35% of AI/ANs are uninsured [49]. Many tribal systems lack electronic medical records [51], and of these systems very few are linked electronically for exchange of information. When possible, linking health data to other registries or tribal documents is tedious and time consuming and may provide another step and opportunity for misclassification.

While health administrators, researchers, and clinicians recognize the data disparities related to poor identification and tracking of AI/AN identities, some systems of care do not consistently collect racial/ethnic identity. In a conference on race and ethnicity data collection held in Portland, Oregon, in 2011, it was reported that misclassification and underreporting of AI/AN health resulted from system and personal related factors, such as (a) data entry error, (b) provider failure to collect the information, (c) patient refusal to provide the information, and (d) patient lack of identification with a specific racial category [48].

Further reasons for misclassification and error in reporting among AI/ANs have been identified and provided in a web-based module from the Native American Cancer Research Center that includes (a) subjective use of personal observations by those recording data, (b) lack of AI/AN as a response category in some medical records, (c) imprecise definitions for AI/AN, (d) changes in status, for example, tribes being formally recognized or unrecognized, (e) tribal enrollment blood percentages changing, (f) tribal enrollment ordinance changes about paternal or maternal blood line lineage, and (g) Spanish surnames leading to AI/ANs categorized as Hispanic [12].

Some researchers have aimed to gain greater understanding of the conditions related to misclassification and suggest that racial stereotypes may be an obstacle [4]. When matching Oklahoma state sexually transmitted infection (STI) surveillance data to the Oklahoma State IHS Patient Registry, misclassification by the surveillance registry was identified and rates for STIs increased among women diagnosed with syphilis by 27% and by 57% for women diagnosed with gonorrhea. Overall, AI/AN women were often misclassified as “White,” and an inverse relationship was found between being reported as “White” and the percentage of AI ancestry [8].

Likewise, age and physical appearance matter. Errors in reporting mortality among AI/ANs in the Pacific Northwest found that both AI/AN men and women had equal chances of being misclassified and younger and older people were more likely to be misclassified as white (19.8% and 17.7%, resp.) as well as those living in urban settings (15.8%) and those with lower blood quantum (less than 25% of Indian blood) (43.6%) [11].

In a study of cirrhosis of the liver and racial classification, the odds of being classified as AI/AN were 2.9 times higher for those who died of cirrhosis [4]. The authors concluded that racial classification may be affected by social processes that shape racial classification and may conform to widely held stereotypes. These disturbing results suggest racial phenotype has an impact on perceived cause of death, which could further confound mortality data for other diseases. In particular, mortality data attribute hepatic disease as the fifth leading cause of death for AI/AN men [52] but if this is an artifact of stereotype, this leaves the true cause of death in question.

### 3.3. Efforts at Correcting Misclassification

Cross-verification, in which registries verify their records with databases of known AI/AN populations, has been shown to capture a more reliable approximation of health [1, 3, 5]. The landmark study by Frost et al. [1] identified a high misclassification of cancer rates among AI/ANs when linking the IHS Registry to the Seattle-Puget Sound Surveillance, Epidemiology, and End Results Registry. Degree of Indian blood quantum affected misclassification, with lower blood quantum being categorized as non-Indian. Similar findings were found in another study that linked tribal enrollment to state cancer registries which found a 97% increase in cancer cases among AI/AN who had been misclassified as non-AI/AN [3].

Puukka et al. [5] compared two methods for calculating cancer incidence, the historical method to two new methods. In the historical method, cancer incidence rate estimates had not incorporated any adjustments for racial misclassified cases. AI/AN patients in the state cancer registries were used as the numerator and the AI/AN specific population in the US census were used as the denominator. In the new (method 1) rates, AI/AN patients with cancer in the state registries had to be verified in the tribal registries and these data were used in the numerator. A record linkage to the tribal registry was conducted. The tribal registry was used as the denominator as well. In method 2, the researchers calculated all the AI/AN with cancer regardless of the verification with the Tribal registry, which was used in the numerator. Both state registries and Tribal Registries were used in the denominator for method 2. They found that the cancer among AI/AN population was considerably higher than had been previously understood using the new methods of calculation.

While linking and cross-verifying data with tribal registries improves data reporting, it is important to note that tribal registries and the IHS database are wrought with challenges. Most importantly, when a tribe makes their registry available for linkage, the criterion for enrollment in that specific tribe (and tribal registry) differs from tribe to tribe, affecting reporting sensitivity among the AI/AN population. Likewise, those individuals who are eligible to receive IHS care are eligible under nonstandardized criteria (variable enrollment criteria among tribes), or may be eligible until age 19 and then no longer, or are eligible because they meet a certain degree of Indian blood but may not be enrolled in a tribe [53, 54]. Consequently, some AI/ANs are discounted. These identified issues are the “tip of the iceberg” for recognizing the complex issue of AI/AN identity.

## 4. Solutions

First and foremost, the best step to minimizing racial and ethnic misclassification is to use the appropriate terminology and understand that in most cases, race is not the most appropriate term. Foster and Sharp [55] have urged scientists to consider what their real question is before selecting race versus ethnicity as a variable. If genomic research is used and race is applied as a geographical location, then perhaps race is the appropriate category [45, 55] if the intent is to gauge the effect of environment on genetics. Likewise, if the intent is to understand health in relation to disparities, then it may be best to look at factors that address socioeconomics, access to health care, and education level, rather than use race or ethnicity.

As has been outlined in this paper, AI/AN ethnicity is varied and complex and ranges from federal and tribally constructed criteria to self-identifying as AI/AN. In facilitating a tribe's rights to exercise sovereignty and facilitate good relations, researchers would benefit from having communities decide on inclusion criteria for AI/AN identity such as enrollment rolls, ancestry, or other specific variables as determined by the tribe. If one is working with IHS, then inclusion criteria may vary and include individuals possessing the certificate of degree of Indian blood, descendants (until they are 19 and can no longer receive care at IHS sites), and/or enrolled tribal members. Large scale studies may benefit from those who self-identify as AI/AN. We recommend that guidance from the AI/AN community is warranted when choosing the population and how it is defined. We also are careful to caution that often AI/AN identity is defined by participation and membership in the tribal or urban/collective group, and research that dissects or removes the AI/AN research subject from the group identity can be seen as contrary to the AI/AN identity as a whole.

Additional recommendations for addressing this problem include (a) continued work on standardization of methods to calculate disease rates, (b) continued work in linking systems of care, state and IHS registries, and tribal enrollment records, (c) greater involvement of patient family members rather than the medical examiner or another health professional's observation in deciding race in death certificates, (d) greater training of health professionals to record race and ethnicity more accurately, (e) greater education of patients on the importance or reporting race and ethnicity, and (f) support for funding tribes to establish computerized tracking systems [3, 5, 11]. A summary of proposed solutions from the research, policy and community perspectives is included in Table 2.

## 5. Conclusions

This paper describes two central problems regarding accuracy in AI/AN health data: (a) misclassification and (b) the complexity in defining AI/AN identity. While linking data to tribal registries (e.g., IHS or tribe specific health care databases) does improve data on AI/AN health disparities, these registries are incomplete. Additionally, common reasons for misclassification among AI/ANs have been identified, as summarized in Table 1. Critical historical events and ongoing political issues that contextualize AI/AN identity have been discussed and must be considered when planning a study or making policy changes. Finally, proposed solutions to mediate the identified misclassification and identity problems among AI/ANs have been presented. The goal of this paper is not to advocate for standardized criteria when identifying the AI/AN population; rather, we advocate for researchers, policy makers, and funding agencies not only to understand this complex identity issue and question how the AI/AN population is being captured, but to also report the criteria used when disseminating findings. Working within the lens of critical race theory, it is our intent not only to invite readers to build an awareness of the complexity of racial misclassification beyond the previously understood solutions of cross-linkage and interagency collaboration, but also to see the issue within the historical context of AI/AN identity, history of governmental policy and its influence on AI/AN identity, and the importance of moving the discourse to the perspective of those living in the margins [37].

In summary, the issue of errors in reporting and misclassification is multifaceted. Researchers and policy makers must be careful not to link socially constructed categories directly to genetics, given our global history of a racialized science [56]. Despite efforts undertaken to transcend discrimination brought about by socially constructed categories of race, we all are shaped by our society wherein race and ethnicity organize our perceptions, relations, and behaviors. We must be aware of our biases to guard against reproducing the same mistakes [56]. This starts with understanding the difference between race and ethnicity, and using the terminology appropriately. We strongly recommend the science community advocate for appropriate use of terminology, to better inform policy makers, accurately identify health disparities, and improve efforts to advocate for resources from funding sources. No other racial or ethnic group in the US must undergo documented scrutiny to “prove” their identity, and it is our hope that information provided in this paper has shed light on the sensitive and complex issue of AI/AN identity.

## Figures and Tables

**Table 1:** Reasons for racial misclassification in American Indian/Alaska Native data*.

Systems level	Policy level	Individual level
(i) No electronic medical record available (ii) Unable to link to other registries (iii) No patient query or lack of race/ethnicity field in data collection (iv) Inadequate definitions of AI/AN identity	(i) Spanish surnames—automatically classified as Hispanic (ii) Physical appearance of individual (iii) Age (iv) Decreasing blood quantum	(i) Refusal to answer (ii) Not able to identify with a particular ethnic or racial identity (iii) Forced to identify one race in forms, so the patient picks the one closest to day to day activities (iv) Previous generation did not enroll

*At the time of this writing, there are additional issues regarding some tribes changing enrollment requirements for tribal members. This issue is broad and complex and beyond the scope of this paper.

**Table 2:** Proposed solutions to racial misclassification.

Domain	Solutions
Research	(i) Standardized methods to calculate disease rates across populations (ii) Strengthened linkages between health care systems and tribal enrollment records, and IHS records (iii) Challenge power differences (iv) Collaborate with health professionals for improved race and ethnicity recording in patient medical records

Policy	(i) Create funding sources to support, build, and develop infrastructure such as tribal disease registries (ii) Challenge power differences (iii) Create policy that unifies and defines demographic categories from a community-oriented perspective (iv) Enact legislation requiring consistent, verifiable, and reliable data from reporting sources, including departments of public health, disease registries, offices of vital records and health statistics, and other data end-points

Community	(i) Collaborate with health systems and IHS to provide linkages for improved data quality purposes (ii) Initiate research driving community priorities to the forefront (iii) Empower and educate community members to report and correct race/ethnicity data in medical records (iv) Challenge power differences
